# *Caenorhabditis elegans* HIF-1 Is Broadly Required for Survival in Hydrogen Sulfide

**DOI:** 10.1534/g3.117.300146

**Published:** 2017-09-08

**Authors:** Irini Topalidou, Dana L. Miller

**Affiliations:** Department of Biochemistry, University of Washington School of Medicine, Seattle, Washington 98195

**Keywords:** hydrogen sulfide, hypoxia, tissue-specific expression

## Abstract

Hydrogen sulfide is common in the environment, and is also endogenously produced by animal cells. Although hydrogen sulfide is often toxic, exposure to low levels of hydrogen sulfide improves outcomes in a variety of mammalian models of ischemia-reperfusion injury. In *Caenorhabditis elegans*, the initial transcriptional response to hydrogen sulfide depends on the *hif-1* transcription factor, and *hif-1* mutant animals die when exposed to hydrogen sulfide. In this study, we use rescue experiments to identify tissues in which *hif-1* is required to survive exposure to hydrogen sulfide. We find that expression of *hif-1* from the *unc-14* promoter is sufficient to survive hydrogen sulfide. Although *unc-14* is generally considered to be a pan-neuronal promoter, we show that it is active in many nonneuronal cells as well. Using other promoters, we show that pan-neuronal expression of *hif-1* is not sufficient to survive exposure to hydrogen sulfide. Our data suggest that *hif-1* is required in many different tissues to direct the essential response to hydrogen sulfide.

Hydrogen sulfide (H_2_S) in the environment is produced by industrial sources and natural sources, including volcanic deposits and anaerobic bacteria (Beauchamp *et al.* 1984). In fact, the gut microbiota produces H_2_S, which can influence the activity of host colonocytes (Beaumont *et al.* 2016). H_2_S is also endogenously produced by animals as a product of cysteine biosynthesis through the transsulfuration pathway, and endogenous H_2_S has important roles in cellular signaling (Li *et al.* 2011; Vandiver and Snyder 2012; Wang 2012). Chronic exposure to relatively low concentrations of environmental H_2_S in humans has been associated with neurological, respiratory, and cardiovascular dysfunction (Kilburn and Warshaw 1995; Richardson 1995; Bates *et al.* 2002). However, transient exposure to low H_2_S has also been shown to improve outcome in many mammalian models of ischemia-reperfusion injury (Bos *et al.* 2015; Wu *et al.* 2015). Although the mechanistic basis of the physiological effects of H_2_S are poorly understood, it is possible that the biological effects of exogenous H_2_S exposure, both beneficial and detrimental, result from activation of pathways that are normally regulated by endogenous H_2_S.

*Caenorhabditis elegans* is an excellent system to define physiological responses to exogenous H_2_S. In addition to powerful genetics, all cells are directly exposed to the gaseous environment (Shen and Powell-Coffman 2003). This feature allows for control of cellular H_2_S exposure without confounding factors from physiological regulation of gas delivery. *C. elegans* grown in 50 ppm H_2_S are long-lived, thermotolerant, and resistant to the hypoxia-induced disruption of proteostasis (Miller and Roth 2007; Fawcett *et al.* 2015). HIF-1 directs the transcriptional response to H_2_S in *C. elegans* (Budde and Roth 2010; Miller *et al.* 2011). HIF-1 is a highly conserved transcription factor best known for regulating the transcriptional response to low oxygen (hypoxia) in metazoans (Semenza 2000, 2001). *C. elegans hif-1* mutant animals are viable and fertile in room air but die if exposed to hypoxia during embryogenesis (Jiang *et al.* 2001; Nystul and Roth 2004). By contrast, exposure to low H_2_S is lethal for *hif-1* mutant animals at all developmental stages (Budde and Roth 2010), and mutations in *hif-1* suppress protective effects of some mutations that confer tolerance to H_2_S (Budde and Roth 2010; Livshits *et al.* 2017). Moreover, increasing the activity of HIF-1, by mutations in negative regulators VHL-1, EGL-9, or RHY-1, increases the tolerance of *C. elegans* to otherwise lethally high concentrations of H_2_S (Budde and Roth 2010; Livshits *et al.* 2017). These observations indicate that HIF-1 has a central role in the organismal response to H_2_S.

Several studies have argued for neuronal-specific functions of HIF-1, although the *hif-1* promoter is active in most, if not all, cells, and HIF-1 protein is stabilized ubiquitously in *C. elegans* exposed to either hypoxia or H_2_S (Jiang *et al.* 2001; Budde and Roth 2010). Neuronal expression of *hif-1* in hypoxia is reported to be sufficient to prevent hypoxia-induced diapause and to increase lifespan through induction of intestinal expression of the flavin monooxygenase FMO-2 (Miller and Roth 2009; Leiser *et al.* 2015). Furthermore, neuronal expression of the cysteine synthase-like protein CYSL-1 regulates the activity of HIF-1 to modulate behavioral responses to changes in oxygen availability (Ma *et al.* 2012). These data motivated us to determine whether neuronal HIF-1 activity is sufficient for *C. elegans* to survive exposure to H_2_S.

In this study, we used tissue-specific rescue of *hif-1* to define the site of essential HIF-1 activity in low H_2_S. We found that expression of *hif-1* from the *unc-14* promoter was sufficient for survival in H_2_S. Although it is considered a pan-neuronal promoter (Ogura *et al.* 1997; Pocock and Hobert 2008), our data indicate that the *unc-14* promoter is also broadly expressed in nonneuronal cells. We show that *hif-1* expressed from the pan-neuronal *rab-3* promoter is not sufficient for viability in H_2_S. We further demonstrate that expression of *hif-1* in muscle, hypodermis, and intestine is not sufficient for viability in low H_2_S. Together, our data indicate that the activity of HIF-1 may be required in multiple tissues to coordinate the organismal response to H_2_S.

## Materials and Methods

### Strains

Strains were grown at room temperature on nematode growth media plates (NGM) seeded with the OP50 strain of *Escherichia coli* (Brenner 1974). All strains were derived from N2 (Bristol). Full genotypes of strains used in this study are shown in Table 1. To sequence the *Punc-14*::*hif-1* junction of *otIs197*, the region was amplified with forward primer oET479 (5′-GTTGTCCACCATCACAGTAATACG-3′) and reverse primer oET480 (5′-ACGACGGCGTTCCATG-3′). The oET479 primer was used for sequencing.

**Table 1:** Strains used in this study

ZG31: *hif-1*(*ia4*) *V*
DLM25: *hif-1*(*ia4*) *V*; *otIs197*[*Punc-14*::*hif-1P621A*, *Pttx-3*::*RFP*]
DLM26: *hif-1*(*ia4*) *V*; *otEx3165*[*Punc-120*::*hif-1P621A*, *Pttx-3*::*RFP*]
XZ2056: *hif-1*(*ia4*) *V*; *yakEx126*[*Punc-17*::*hif-1cDNA*, *Pmyo-2*::*mCherry*]
XZ2065: *hif-1*(*ia4*) *V*; *yakEx131*[*eef-1A.1*::*hif-1cDNA*, *Pmyo-2*::*mCherry*]
XZ2073: *hif-1*(*ia4*) *V*; *yakEx137*[*Punc-14*::*hif-1P621A*::*YFP*, *Pmyo-2*::*mCherry*]
XZ2074: *hif-1*(*ia4*) *V*; *yakEx136*[*Pvha-6*::*hif-1cDNA*, *Pmyo-2*::*mCherry*]
XZ2080: *yakEx142*[*Punc-14*::*GFP*, *Pmyo-2*::*mCherry*]
XZ2081: *hif-1*(*ia4*) *V*; *yakEx143*[*Pdpy-7*::*hif-1cDNA*, *Pmyo-2*::*mCherry*]
XZ2082: *hif-1*(*ia4*) *V*; *yakEx144*[*Punc-14*::*hif-1cDNA*, *Pmyo-2*::*mCherry*]
XZ2083: *hif-1*(*ia4*) *V*; *yakEx145*[*Punc-47*::*hif-1cDNA*, *Pmyo-2*::*mCherry*]
XZ2084: *hif-1*(*ia4*) *V* ; *yakEx125*[*Prab-3*::*hif-1cDNA*, *Pmyo-2*::*mCherry*]
XZ2085: *hif-1*(*ia4*) *V* ; *yakEx146*[*Pvha-6*::*hif-1cDNA*, *Pdpy-7*::*hif-1cDNA*, *Prab-3*::*hif-1cDNA*, *Pmyo-2*::*mCherry*]

### Constructs and transgenes

All constructs were made using the multisite Gateway system (Invitrogen), where a promoter region, a gene region (*hif-1* cDNA or GFP), and a C-terminal 3′ untranslated region (UTR) were cloned into the destination vector pCFJ150 (Frøkjær-Jensen *et al.* 2008). The *hif-1* A isoform was amplified from cDNA using forward primer oET467 (5′-GGGGACAAGTTTGTACAAAAAAGCAGGCTCAATGGAAGACAATCGGAAAAGAAAC-3′) and reverse primer oET469 (5′-GGGGACCACTTTGTACAAGAAAGCTGGGTGTCAAGAGAGCATTGGAAATGGG-3′). For the tissue-specific rescuing experiments, an operon GFP::H2B was included in the expression constructs downstream of the 3′UTR (Frøkjær-Jensen *et al.* 2012). This resulted in expression of untagged HIF-1 protein and histone H2B fused to GFP, which allowed for confirmation of promoter expression by monitoring GFP expression. The *unc-14* promoter (1425 bp upstream of the start codon) was amplified from genomic DNA using forward primer oET520 (5′-GGGGACAACTTTGTATAGAAAAGTTGGAGAGCAGCAGCATCTCGAG-3′) and reverse primer oET507 (5′-GGGGACTGCTTTTTTGTACAAACTTGTTTTGGTGGAAGAATTGAGGG-3′). All plasmids constructed were verified by sequencing. Constructs used in this study are shown in Table 2. Extrachromosomal arrays were made by standard injection methods (Mello *et al.* 1991) with 10–15 ng/μl of the expression vector. At least two independent lines were isolated for each construct.

**Table 2:** Plasmids and constructs used in this study

Gateway entry clones	
pCFJ326	*tbb-2 3′UTR*::OPERON::GFP [2-3]
pCFJ386	*eef-1A.1* [4-1] 625 bp upstream of and including the ATG
pCR110	GFP [1-2]
pEGB05	*Prab-3* [4-1] 1232 bp upstream of the ATG
pET168	*hif-1* cDNA A isoform [1-2]
pET210	*Punc-14* [4-1] 1425 bp upstream of the ATG
pGH1	*Punc-17* [4-1] 3229 bp upstream of and including the ATG
pMH522	*Punc-47* [4-1] 1254 bp upstream of and including the ATG
pET187	*Pdpy-7* [4-1] 350 bp upstream of and including the ATG
pET188	*Pvha-6* [4-1] 881 bp upstream of and including the ATG
Gateway expression constructs	
pET171	*Punc-47*::*hif-1 cDNA*::*tbb-2 3′UTR*::OPERON::GFP_pCFJ150
pET172	*Punc-17*::*hif-1 cDNA*::*tbb-2 3′UTR*::OPERON::GFP_pCFJ150
pET182	*Prab-3*::*hif-1 cDNA:tbb-2 3′UTR*::OPERON::GFP_pCFJ150
pET187	*Pdpy-7*::*hif-1 cDNA*::*tbb-2 3′UTR*::OPERON::GFP_pCFJ150
pET188	*Pvha-6*::*hif-1 cDNA*::*tbb-2 3′UTR*::OPERON::GFP_pCFJ150
pET212	*Punc-14*::*GFP*::*let-858 3′UTR*_pCFJ150
pET213	*Punc-14*::*hif-1 cDNA*::*tbb-2 3′UTR*::OPERON::GFP_pCFJ150
pET216	*eef-1A.1*::*hif-1 cDNA*::*tbb-2 3′UTR*::OPERON::GFP_pCFJ150

### H_2_S atmospheres

Construction of atmospheric chambers was as previously described (Miller and Roth 2007; Fawcett *et al.* 2012). In short, H_2_S (5000 ppm with balance N_2_) was diluted continuously with room air to a final concentration of 50 ppm. Final H_2_S concentration was monitored using a custom-built H_2_S detector containing a three-electrode electrochemical SureCell H_2_S detector (Sixth Sense) as described (Miller and Roth 2007), calibrated with 100 ppm H_2_S with balance N_2_. Compressed gas mixtures were obtained from Airgas (Radnor, PA) and certified as standard to within 2% of the indicated concentration. H_2_S atmospheres were maintained at 20°.

### Survival assays

20 to 40 L4 animals were picked to plates seeded with OP50. Plates were exposed to 50 ppm H_2_S for 20–24 hr in a 20° incubator, and then returned to room air to score viability. Death was defined as failure to move when probed with a platinum wire on the head or tail. Animals were scored 30 min after removal from H_2_S, and plates with dead animals were reexamined after several hours to ensure animals had not reanimated.

### Imaging

For imaging expression of GFP, larval stage 1 (L1) or first-day adult animals were mounted on 2% agarose pads and anesthetized with 50 mM sodium azide for 10 min before placing the cover slip. The images were obtained using a Nikon 80i wide-field compound microscope.

### Data availability

Strains are available upon request and have been deposited at the *Caenorhabditis* Genetics Center (cgc.umn.edu). Plasmid constructs are available upon request.

## Results and Discussion

**Figure 1:**
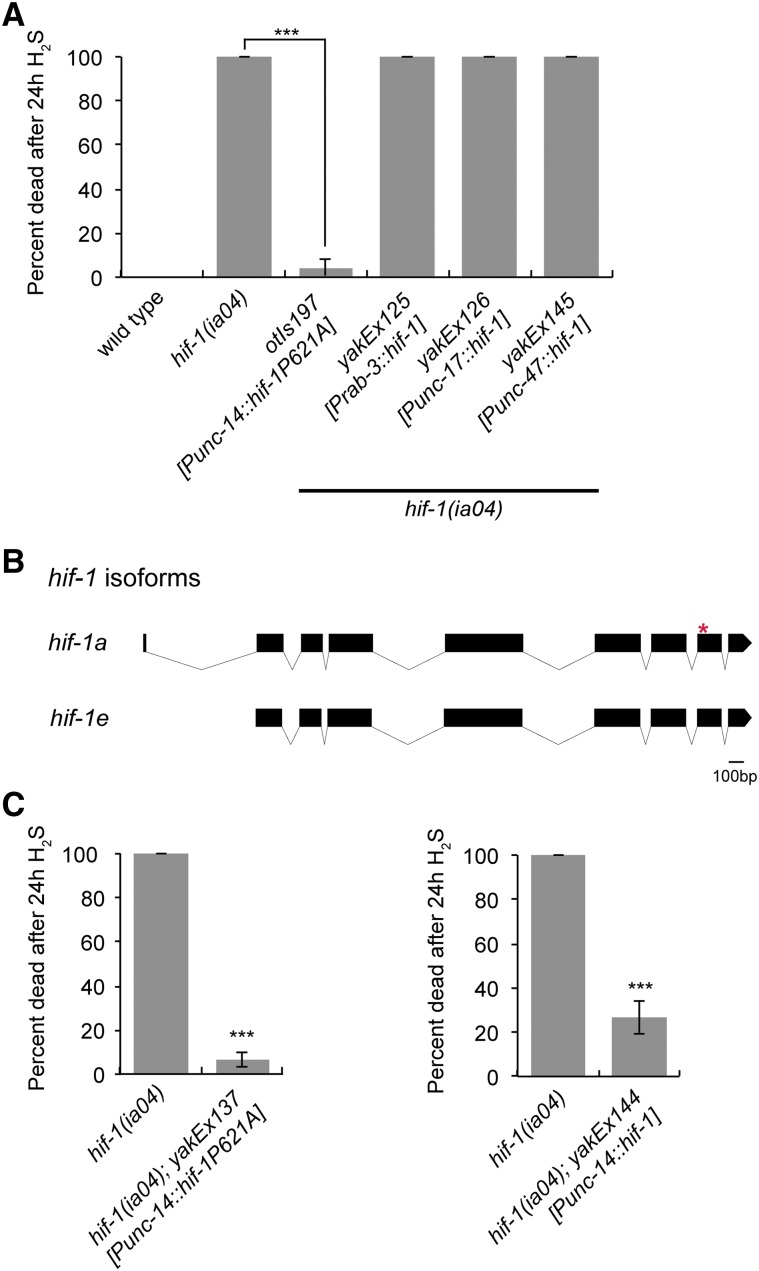
HIF-1 expression from the *unc-14* promoter rescues the H_2_S lethality of *hif-1*(*ia4*) mutant animals. (A) Survival of animals exposed to H_2_S. All animals have the null *hif-1*(*ia4*) mutation. The *otIs197* integrated array expresses a nondegradable HIF-1 variant. Other constructs were extrachromosomal arrays that express wild-type HIF-1. The *unc-14* promoter is expressed pan-neuronally (Ogura *et al.* 1997), the *rab-3* promoter is expressed in most, if not all, neurons (Nonet *et al.* 1997), *unc-17* is expressed in cholinergic neurons (Rand *et al.* 2000), and *unc-47* is expressed in GABAergic neurons (Eastman *et al.* 1999). Animals were exposed to 50 ppm H_2_S starting at L4. (B) HIF-1 gene structure and predicted A and E isoforms (Wormbase 2017). The P621A mutation that prevents degradation of *hif-1* included in *otIs197* is marked with *. (C) Survival of animals expressing HIF-1 from *unc-14* promoter exposed to H_2_S. All animals have the null *hif-1*(*ia4*) mutation. Expression of HIF-1 was from extrachromosomal arrays. The *yakEx137* array expresses nondegradable HIF-1(P621A) and the *yakEx144* array expresses wild-type *hif-1*. For all panels, animals were exposed to 50 ppm H_2_S starting at L4. Average of three independent experiments is shown, each with *n* = 20–40 animals. Error bars are SEM. In all panels, statistical comparisons were to *hif-1*(*ia4*) controls. Statistically significant differences are indicated with *** *P* < 0.001 (Fisher’s exact test).

*C. elegans* requires *hif-1* to survive exposure to low H_2_S (Budde and Roth 2010). To determine whether neuronal expression of *hif-1* was sufficient for survival in H_2_S, we used transgenic *hif-1*(*ia4*) mutant animals that expressed *hif-1* from heterologous promoters. We first used the available *otIs197* transgene, which expresses *hif-1* from the putative pan-neuronal *unc-14* promoter (Pocock and Hobert 2008). We found that *hif-1*(*ia4*); *otIs197* animals survived exposure to 50 ppm H_2_S (Figure 1A). This result suggests that neuronal expression of *hif-1*, from the *unc-14* promoter, is sufficient to survive exposure to H_2_S.

To further dissect in which neuronal cell type(s) HIF-1 activity was required to survive exposure to H_2_S, we generated transgenic animals that expressed *hif-1* cDNA under the control of promoters active in specific neuronal subtypes. We found that expression in neither cholinergic neurons (*Punc-17*) nor GABAergic neurons (*Punc-47*) was sufficient to rescue the lethality of *hif-1*(*ia4*) mutant animals exposed to H_2_S (Figure 1A). Curiously, we also observed that expression of *hif-1* cDNA from the pan-neuronal *rab-3* promoter did not rescue survival of the *hif-1*(*ia4*) mutant animals (Figure 1A). This was unexpected, as expression of HIF-1 from the *unc-14* promoter (the *otIs197* transgene) was sufficient for survival in H_2_S. We therefore pursued the source of this discrepancy.

We first sought to verify the molecular nature of the *otIs197* integrated transgene. We used PCR to amplify a region from the *unc-14* promoter and the *hif-1* coding region from the *otIs197* transgenic animals. As expected, this reaction generated a single band of approximately 500 bp. However, when we sequenced the resulting PCR product, we discovered an insertion of an extra G immediately following the ATG of the *hif-1* cDNA. This insertion causes a frame-shift and results in a stop codon after 13 amino acids. However, the *otIs197* transgene must express some HIF-1 protein, as it can rescue many phenotypes of *hif-1* mutant animals (Pocock and Hobert 2008; Miller and Roth 2009; Ma *et al.* 2012; Leiser *et al.* 2015). The *otIs197* transgene was constructed to express isoform A of *hif-1*, though there are six predicted isoforms (WormBase 2017). We noted that the ATG for isoform E is 21 bp downstream of the original ATG in the *hif-1* cDNA. Thus, it could be that expression of the *hif-1e* isoform is the basis of the activity of the *otIs197* transgene. Because our *Prab-3*::*hif-1* transgene expressed the *hif-1a* isoform, it was possible that the differences we observed from *otIs197* were due to the expression of different *hif-1* isoforms. To test this possibility we created transgenic strains expressing *hif-1a* under control of the *unc-14* promoter using a *Punc-14*::*hif-1a*(*P621A*)::*YFP* plasmid (Pocock and Hobert 2008), which we verified had had the expected *hif-1a*(*P621A*) sequence. We injected this plasmid into *hif-1*(*ia4*) mutant animals to generate the *yakEx137* transgene. If the rescue we observed in *otIs197* was due to expression of *hif1e* rather than *hif1a*, then the animals expressing *Punc-14*::*hif-1a*(*P621A*)::*YFP* would die in H_2_S. However, these animals survived exposure to H_2_S (Figure 1C), indicating that potential expression of different isoforms did not underlie differences in survival of exposure to H_2_S.

The HIF-1 protein expressed by the *otIs197* transgene has a P621A mutation that prevents it from being hydroxylated and degraded by the proteasome (Pocock and Hobert 2008). By contrast, the constructs we generated produced wild-type HIF-1 protein. We did not expect this feature to be salient for our experiments, since HIF-1 protein is stabilized in H_2_S due to inhibition of the hydroxylation reaction (Budde and Roth 2010; Ma *et al.* 2012). However, it is possible that constitutive stabilization of HIF-1 protein in neurons promotes survival in H_2_S. To evaluate this possibility, we cloned wild-type *hif-1* cDNA under control of the *unc-14* promoter, including 1.4 kb upstream of the transcription start site (Ogura *et al.* 1997). We found that *hif-1*(*ia4*); *Punc-14*::*hif-1* (*yakEx144*) animals survived exposure to H_2_S, similar to *hif-1*(*ia4*); *otIs197* animals (Figure 1C). We conclude that the P621A mutation in *otIs197* does not underlie the difference in survival in H_2_S that we observed for animals expressing *hif-1* from *rab-3* and *unc-14* promoters.

**Figure 2:**
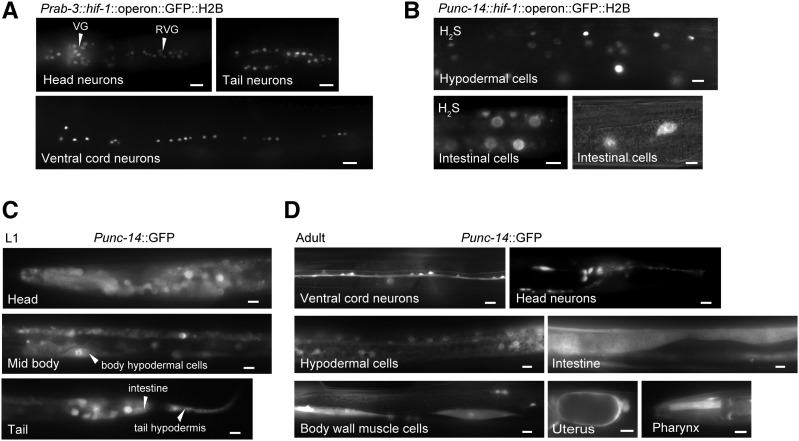
The *unc-14* promoter is active in many nonneuronal cells. (A) Visualization of GFP expressed from *Prab-3*::*hif-1*::*operon*::*GFP*::*H2B* (transgene *yakEx125*). Tail, head, and ventral cord neurons are shown from the ventral aspect of the same animal. VG, ventral ganglia; RVG, retrovesicular ganglia. In all images: bar, 10 μm. (B) Representative images of adult *hif-1*(*ia4*); *Punc-14*::*hif-1*::*operon*::*GFP*::*H2B* (transgene *yakEx144*) animals. GFP expression in hypodermal and intestinal cells is shown. Bar, 10 μm. (C and D) Representative images of (C) L1 and (D) adult transgenic animals expressing *Punc-14*::*GFP* (transgene *yakEx142*). Representative animals are shown with GFP expression in hypodermis, intestine, muscle, uterus, pharynx, and neurons. Bar, 5 μm in (C) and 10 μm in (D).

Given that the only other notable difference between the *Prab-3*::*hif-1* and *Punc-14*::*hif-1* constructs is the promoter elements, we hypothesized that differences between either the levels of expression from these promoters or the identity of the cells where these promoters are expressed should account for their different behavior. The transgenic constructs we generated all included an operon GFP::H2B downstream of the 3′UTR (Frøkjær-Jensen *et al.* 2012). This resulted in expression of untagged HIF-1 protein as well as GFP::H2B. We therefore visualized GFP expression to evaluate the expression levels and cellular patterns of promoter activity. As expected, GFP expression from adult *hif-1*(*ia4*); *Prab-3*::*hif-1*::*operon*::*GFP*::*H2B* was exclusively in neurons (Figure 2A). However, when we imaged adult *hif-1*(*ia4*); *Punc-14*::*hif-1*::*operon*::*GFP*::*H2B* animals that had survived exposure to H_2_S, we observed GFP expression in neurons, as expected, but also in intestinal and hypodermal cells (Figure 2B). We saw similar expression in animals that had not been exposed to H_2_S. To corroborate this observation, we cloned the *unc-14* promoter upstream of GFP and injected it into wild-type animals. We then imaged larvae (Figure 2C) and adult animals (Figure 2D) from three separate lines. We observed expression of GFP in numerous cells other than neurons including intestine, hypodermis, muscle, and the uterus. Every animal that we imaged had expression in at least one cell type other than neurons (*n* = 50).

**Figure 3:**
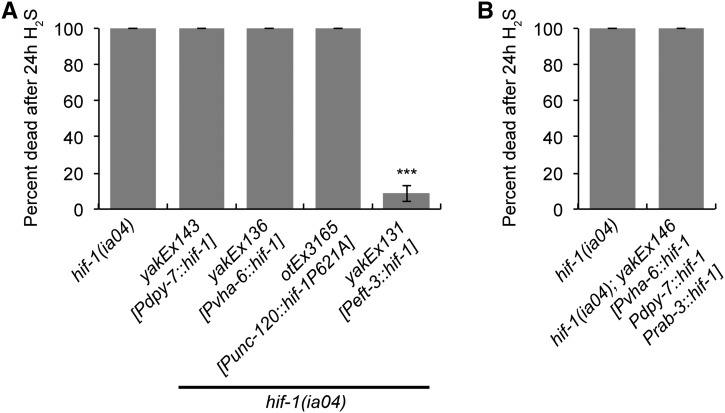
Survival in H_2_S requires broad expression of *hif-1*. Survival of animals exposed to H_2_S. All animals have the null *hif-1*(*ia4*) mutation. (A) Lethality of animals that express *hif-1* only in hypodermis (*Pdpy-7*::*hif-1*; *yakEx143*), intestine (*Pvha-6*::*hif-1*; *yakEx136*), or muscle (*Punc-120*::*hif-1*(*P621A*); *otEx3165*). As a control, *hif-1* was expressed from a ubiquitous promoter (*eef-1A.1*::*hif-1*; *yakEx131*). Expression was from extrachromosomal arrays. Wild-type *hif-1* was used for all constructs except the *Punc-120*::*hif-1*(*P621A*), which expresses the nondegradable variant. (B) Survival of *hif-1*(*ia4*); *yakEx146* animals exposed to H_2_S that express *hif-1* simultaneously in intestine (*Pvha-6*::*hif-1*), hypodermis (*Pdpy-7*::*hif-1*), and neurons (*Prab-3*::*hif-1*). Average of three independent experiments is shown, each with *n* = 20–35 animals. Error bars are SEM. In all panels, statistical comparisons were to *hif-1*(*ia4*) controls. Statistically significant differences are indicated with *** *P* < 0.001 (Fisher’s exact test).

Based on our understanding of *Punc-14* expression and the fact that *hif-1*(*ia4*); *Prab-3*::*hif-1* animals die when exposed to H_2_S (Figure 1A), we inferred that neuronal HIF-1 activity is not sufficient for survival in H_2_S. We therefore explored whether expression of *hif-1* exclusively in nonneuronal tissues was sufficient for survival in H_2_S. For these experiments, we generated transgenes with *hif-1* expressed under control of the *unc-120* promoter, which is active in body-wall and vulval muscle; the *dpy-7* promoter, which is active in hypodermis; the *vha-6* promoter, which is active in intestine; and the ubiquitous *eef-1A.1* promoter. We chose these promoters because they included many of the tissues that had *unc-14*-driven expression of GFP (Figure 2B). As shown in Figure 3, only the ubiquitously expressed *eef-1A.1*::*hif-1* rescued the lethality of *hif-1*(*ia4*) mutants exposed to H_2_S. Although we did not test all possible cell and tissue types, these data suggest that HIF-1 activity in a single tissue cannot support survival in H_2_S.

The fact that *Punc-14*::*hif-1* was sufficient for survival in H_2_S (Figure 1A) suggests that activity of HIF-1 may not be required in all cells. Since we did not observe rescue when *hif-1* was expressed in a single tissue, we made transgenic animals with expression of *hif-1* in >1 tissue to determine whether we could find a minimal expression that was sufficient for survival in H_2_S. We found that even animals with *hif-1* expression in neurons, hypodermis, and intestine—*hif-1*(*ia4*); *yakEx146*[*Prab-3*::*hif-1*, *Pvha-6*::*hif-1*, *Pdpy-7*::*hif-1*]—did not survive exposure to H_2_S (Figure 3B). Together, our data suggest that that HIF-1 activity is required in many tissues to coordinate the essential response to H_2_S. This could indicate that HIF-1 acts cell-autonomously to direct expression of many tissue-specific transcripts that are required to survive exposure to H_2_S.

Although it was reported that *otIs197* expresses *hif-1* selectively in neurons (Pocock and Hobert 2008), our data show that the *unc-14* promoter is more broadly expressed. In fact, others have reported nonneuronal expression of transgenes expressed under the control of the *unc-14* promoter (Ogura *et al.* 1997; Wolkow *et al.* 2000; da Graca *et al.* 2004). However, the nonneuronal expression we have demonstrated is much more penetrant than has been previously acknowledged. This is an important consideration when interpreting the results of experiments using transgenes driven by *unc-14*, including *hif-1* from *otIs197*. Our data show that nonneuronal expression from the *unc-14* promoter is significant, and that rescue by *unc-14*-driven transgenes is not sufficient to infer neuronal function of HIF-1 and, presumably, other proteins.
